# Association between co-sleeping in the first year of life and preschoolers´ sleep patterns

**DOI:** 10.1007/s00431-024-05429-2

**Published:** 2024-02-14

**Authors:** Felipe Garrido, Juan-Luis González-Caballero, Pilar García, Maria-Lorella Gianni, Silvia Garrido, Lucía González, Verónica Atance, Genny Raffaeli, Giacomo Cavallaro

**Affiliations:** 1 Department of Pediatrics, Clínica Universidad de Navarra. Calle Marquesado de Santa Marta, 1 Madrid (28227), Spain; 2 Department of Statistics and Operations Research, Faculty of Medicine, University of Cadiz, 11003 Cádiz, Spain; 3 Jerome Lejeune Institute, Madrid (28227), Spain; 4 Department of Clinical Sciences and Community Health, Università degli Studi di Milano, 20122 Milan, Italy; 5 Neonatal Intensive Care Unit. Fondazione IRCCS Ca’ Granda Ospedale Maggiore Policlinico. 20122, Milan, Italy; 6 Department of Pediatrics, Clínica Universidad de Navarra, Madrid (28227), Spain

**Keywords:** Co-sleeping, Sleep pattern, Preschool Children, Infants

## Abstract

This study aimed to investigate the association between co-sleeping practiced during the first year of life and preschoolers' sleep patterns. A cross-sectional study including toddlers was designed to analyze their sleep patterns. The Brief Infant Sleep Questionnaire, validated in Spanish, was used to measure sleep quality. A latent class analysis was performed to identify qualitative subgroups in the sample and explore the effects of co-sleeping. The sleep patterns of 276 children were analyzed. A total of 181 (65%) parents reported having practiced co-sleeping with their children. The latent class analysis identified a two-class solution with two different sleep patterns. One of them showed a worse quality sleep pattern, which had a significant association with having practiced co-sleeping during the first year of life, and with the fact that they were still sleeping in the parents' room, among other characteristics related to co-sleeping and parental concerns. Breastfeeding also showed association with a worse quality sleep pattern.

*Conclusion*: Based on the present findings, co-sleeping during the first year of life appears to be associated with poor sleep patterns in young preschoolers.

**Table:** 

**What is Known:** *• Co-sleeping shows benefits for infants and parents, mainly facilitating successful breastfeeding.* *• Literature on the effect of co-sleeping in lately sleep quality in children and their parents is very limited.*	
**What is New:** *• Co-sleeping practiced during the first year of life could be associated with a worse sleep pattern measured with BISQ-E tool.* *• A balance between the correct practice of co-sleeping and the achievement of a healthy sleep routine in preschool should probably be part of parents' health education.*

**Supplementary Information:**

The online version contains supplementary material available at 10.1007/s00431-024-05429-2.

## Introduction

Sleep patterns and behaviors of children are influenced by several factors [1, 2]. Establishing a healthy sleep pattern during the first year of life and the early preschool period contributes to optimal development [3]. Sleep difficulties impact health outcomes, including obesity risk, and are among the most frequent reasons for consultation in clinical practice [4, 5].

Co-sleeping shows benefits for infants, facilitating successful breastfeeding [6, 7]. Moreover, from an anthropological point of view, co-sleeping positively modulates affective and psychomotor development [8].

However, co-sleeping has been questioned due to a potential increased risk of sudden infant death, particularly associated with unsafe sleeping practices (i.e., parental smoking, recent alcohol consumption, and sofa sharing). According to the American Academy of Pediatrics (AAP) recommendation, co-sleeping in the same bedroom, next to the parent’s bed but on a specific surface, reduces the risk of sudden infant death syndrome by up to 50% [9]. On the other hand, this risk is increased when babies sleep alone in the same bed as their parents [9, 10].

Studies investigating the relationship between co-sleeping and sleep patterns in infancy have reported inconsistent results indicating any association or the occurrence of more awakenings and prolonged sleep in lighter stages [11, 12]. Knowledge concerning the effect of co-sleeping on sleep patterns lately in infancy is scarce [1].

Our study aimed to investigate the association of co-sleeping, practiced during the first year of life, with the sleep pattern during the early years of preschoolers. The hypothesis to be tested was that co-sleeping during the first year of life would not be associated with preschoolers' sleep patterns.

## Methods

A descriptive, observational, and cross-sectional study was conducted, including a convenient sample of parents of preschoolers aged 12–30 months. The Ethics Committee of the University of Navarra approved the study (code 2022.025). Recruitment of participants was carried out through the outpatient clinics of pediatric health care centers and children's daycare centers, mainly in the regions of Madrid and Valencia (Spain). Parents were invited to participate in primary care centers mainly during health visits and by taking advantage of the routine chicken-pox vaccination, which is scheduled at 15 months of age. There was no prior selection of parents; they were offered to participate, and then written information was provided and posteriorly linked to the survey via a QR code. Parents in daycare centers were offered to participate through information provided to parents through school nurses. Once offered, parents answered the survey without the supervision of any medical/research staff. Parents were recruited for the survey, and data was collected from January to June 2023.

Data was collected through an online questionnaire designed in Spanish, available on the SurveyMonkey^®^ platform. The questionnaire included an introductory part of the study and a total of 32 questions, requiring approximately 10 min to be answered. All parents participated voluntarily and completed an informed consent before answering the questions.

The first part of the questionnaire was designed using the modified Delphi method (22 questions). An online and anonymous reactive Delphi technique was used, where three authors (PG, FG, JLG) with expertise in pediatrics, neurodevelopment, and epidemiology autonomously modified a previously prepared survey (11–14). Consensus on a final version of the survey was reached after three rounds in which researchers proposed modifications to proposed questions and possible answers. The corrections made by each of the members of the survey development were shared and reviewed anonymously.

In the first part of the questionnaire, demographic data, including parents’ age, date of birth and sex of the preschooler, date of answering the questionnaire, and number of children, were collected. Parents were asked whether they commonly used to practice co-sleeping. Common practice of co-sleeping was defined as co-sleeping at least 3 times a week. Co-sleeping was defined for parents as sharing their bed, usually with their children, during sleeping periods. The definition of co-sleeping was also applied to cases where parents shared their bed using a specific co-sleeping cot. If the answer was yes, the following question was related to the time (in months) co-sleeping had been practiced. The remaining questions about co-sleeping and information sheet for parents can be consulted in the Supplementary material. Parents were also asked whether their babies had been breastfed and how long.

The second part of the questionnaire contained the BISQ-E, based on the BISQ (Brief Infant Sleep Questionnaire) [13]. The BISQ is a questionnaire widely used in the literature and validated to assess children´s sleep between 6 and 36 months. The BISQ-E has been validated in Spanish for children between 6 and 30 months and includes questions about parental reports on infant daytime and night-time sleep patterns during the previous two weeks. The validity of BISQ-E has been established by analyzing its correlation with a sleep diary [5]. The sleep diary is a valid and independent instrument for assessing sleep patterns in children and is considered a 'gold standard' instrument for the subjective study of sleep characteristics [14].

### Statistical analysis

We have calculated frequencies and percentages for qualitative variables, and the usual parameters of mean, median, standard deviation, minimum and maximum for quantitative variables. For the latter, we also tested for normality with the Kolmogorov-Smirnov test.

We have carried out a latent class analysis (LCA) using all the items from the BISQ-E questionnaire as indicators, but the one referring to the position where the child sleeps [15]. This model allowed us to classify children into patterns characterized by the indicators [16]. First, we explored the solution with one pattern of initial fit parameters. Then, we increased the number of classes until we obtained the best fit for the model. To evaluate the fit, we used several criteria. First, the Akaike Information Criteria (AIC), the Bayesian Information Criterion (BIC), and the Adjusted BIC, where small values of each indicate a better model fit [17–19]. In the second place, we used the entropy value, a value comprised between 0 and 1, which measures the uncertainty of the classification obtained for the classes, where values greater than 0.80 indicate a solid separation to identify separate groups [20]. In third place, we also used the Lo-Mendell-Rubin Adjusted Likelihood Ratio Test (LMRT) and the Bootstrapped Likelihood-Ratio Test (BLRT) [21], suggesting that, for a p < 0.05, the model with more classes fits the data better than the model with fewer classes. In addition to the statistical fit, we also used the significance and interpretation of the patterns, as well as the probability of the presence of each indicator in the pattern [22].

To perform the latent class analysis, we used the items recoded as binary indicators. Once the profiles were obtained, we calculated the class membership probabilities for everyone. This allowed the identification of a categorical variable within categories by assigning the class with the highest membership probability to each participant. This variable allowed investigation of the association between patterns and other characteristics of parents and children not contained in the BISQ-E questionnaire, using chi-square and t-student tests.

We used Mplus software, version 8.10 (Muthén & Muthén, 3463 Stoner Avenue, Los Angeles, CA 90066, US), to perform LCA [23] and SPSS, version 29 (IBM Corp., Armonk, NY 10504, US) to perform the descriptive analyses and assess the associations between children characteristics and sleep patterns. In all analyses, we considered a p < 0.05 statistically significant.

## Results

A total of 451 parents responded to the survey. The answers of 175 parents had to be discarded because they were either incomplete or related to preschoolers under 12 or over 30 months. The responses of 276 parents were finally analyzed, although we only included in the full analysis 244 cases in which there were responses of at least 8 of the 10 BISQ-E items. The survey was mainly responded to by mothers (243; 88%). The mean maternal age was 35.2 ± 4.4 years. The mean preschoolers' age was 20.4 ± 5.3 months when their parents participated in the survey. More than half of the parents (65%) did not report any sleeping difficulties of their children when the survey was performed. In contrast, nearly one out of two (56%) reported their children had experienced sleeping difficulties in the past. A total of 181 (66%) parents reported having practiced co-sleeping with their children. The median reported time of having practiced co-sleeping was 17 months (IQR 8–24). The demographic data of the sample is shown in Table 1.

**Table 1:** Demographic summary of participants in the survey

**Demographic variables**	**Result (n = 276)**
Mother´s age (years) - mean (SD)	35.2 (4.4)
Father´s age (years) - mean (SD)	37.8 (5.4)
Infant age (months) - mean (SD)	20.4 (5.3)
Sex of infant - n (%):	
- Female - Male	127 (46) 149 (54)
Relevant disease that has caused concern in parents - n (%):	
- Yes - No	15 (5) 261 (95)
Infants´ age transferred to his/her room (in months) - mean (SD):	9 (5)
Parents worried about their infant sleep pattern - n (%)	
- Yes - No	91 (33) 185 (67)
Current difficulties with sleep pattern of infant - n (%)	
- Yes - No	97 (35) 179 (65)
Parent difficulties with sleep pattern of infant - n (%)	
- Yes - No	154 (56) 122 (44)
Co-sleeping practiced during the first year of life (more than 3 times a week) - n (%)	
- Yes - No	191 (69) 95 (31)
Up to what age co-sleeping was practiced (in months): - median (IQR)	17 (8–24)
Planned to practice co-sleeping or started practicing it due to baby requirements - n (%):	
- Planning before birth - Started due to baby requirements	95 (47) 107 (53)
Agreement between parents about co-sleeping - n (%)	
- Yes - No	176 (93) 14 (7)
Infant has been breastfed - n (%)	
- Yes - No	241 (87) 35 (13)
Age of completion of breastfeeding (in months) - median (IQR)	12 (6–18)

Infant age refers to current age. Quantitative variables are summarized as mean (SD) or median and interquartile range (IQR). Rest of categorical variables are expressed as n (%)

The summary of the results of the BISQ-E questionnaire is shown in Table 2. Half of the preschoolers did not have siblings (49%), and in one-third of the cases, the preschooler was the youngest of the family (29%). Fifty-nine (21%) of preschoolers slept in their parents' beds or a cot but in their parents' rooms (19%). The rest slept in their bedroom (48%). The position in which they slept was variable (Table 2). The average night-time sleep time was 10.1 ± 1.2 h, while the daytime sleep time (nap) was 2.1 ± 0.7 h. The median number of nocturnal awakenings was 2 (IQR 1–3). The times (expressed in minutes) to fall asleep at night or to fall asleep once awakened are reported in Table 2. Many preschoolers fell asleep while eating (20%) or in their cot or bed in a room different from their parents (32%). Almost 15% needed to fall asleep in their parents' arms, and 10% in their parents' bed. More than a third of those surveyed reported children fell asleep between 8 and 10 PM.

**Table 2:** Results corresponding to the BISQ-E questionnaire

		**Recoding of items for LCA**
Place where the infant sleeps now (n = 244) - n (%): 1. Sleeps in the cot/bed, in a separate room 2. Sleeps in the cot/bed, in a shared room with another sibling 3. Sleeps in the crib/bed, in the parents' room 4. Sleeps in parents´ bed	107 (39) 25 (9) 53 (19) 59 (21)	0. Sleeping with parents (3,4) 1. Not sleeping with parents (1,2)
Position in which the infant sleeps most of the time (n = 236) - n (%): - On his tummy - On his back - On his side - Not on a define position	62 (22) 49 (18) 50 (18) 75 (27)	
How do you get the infant to fall asleep? (n = 239) - n (%) 1. Falls asleep while eating 2. In his/her cot/bed in a separate room 3. In his/her cot/bed in the parents' room 4. Rocking him in his/her cot or similar 5. Rocking him/her in parents’ arms 6. Holding him/her in the arms without rocking 7. In the same parent's bed	55 (20) 88 (32) 14 (5) 13 (5) 16 (6) 25 (9) 28 (10)	0. Not sleeping in own bed (the rest) 1. Sleeping in own bed (2,3)
During weekdays, at what time does your infant usually fall asleep at night? (n = 244) - n (%) 1. Between 6 PM-8PM 2. Between 8 PM-10 PM 3. Between 10 PM-12 PM	11 (4) 184 (67) 49 (18)	0. To sleep after 10 pm (3) 1. To sleep before 10 pm (1,2)
Do you consider your infant's sleep habits a problem? (n = 216) - n (%) 1. Yes, a very serious problem 2. Yes, although a minor problem 3. No, it's not a problem	35 (13) 52 (19) 129 (47)	0. Sleep is an important issue (1) 1. Sleep is not an important issue (2,3)
Sleep time overnight (hours) (n = 244) – median (IQR)	10.5 (10–11)	0. Sleeping less than 10 h at night 1. Sleeping 10 h or more at night
Sleep time during the day (hours) (n = 244) – median (IQR)	2.2 (2–3)	0. Daytime sleep less than 2 h 1. Daytime sleep 2 h or more
Number of awakenings overnight (n = 244) – median (IQR)	2 (1–4)	0. Two or more night-awakenings 1. Less than two night-awakenings
Awake time overnight (minutes) (n = 244) – median (IQR)	9 (0–45)	0. Awake time overnight more than 10 min 1. Awake time overnight 10 min or less
Time it takes to fall sleep (minutes) (n = 244) – median (IQR)	20 (10–20)	0. Time to get sleep more than 20 min 1. Time to get sleep 20 min or less

The results of the categorical variables are expressed with their frequencies and percentages. The results of the quantitative variables are expressed by calculating the medians, and interquartile ranges (IQR 25–75). Variables have been recoded into dichotomic values to perform LCA (Latent Class Analysis)

**Fig. 1:**
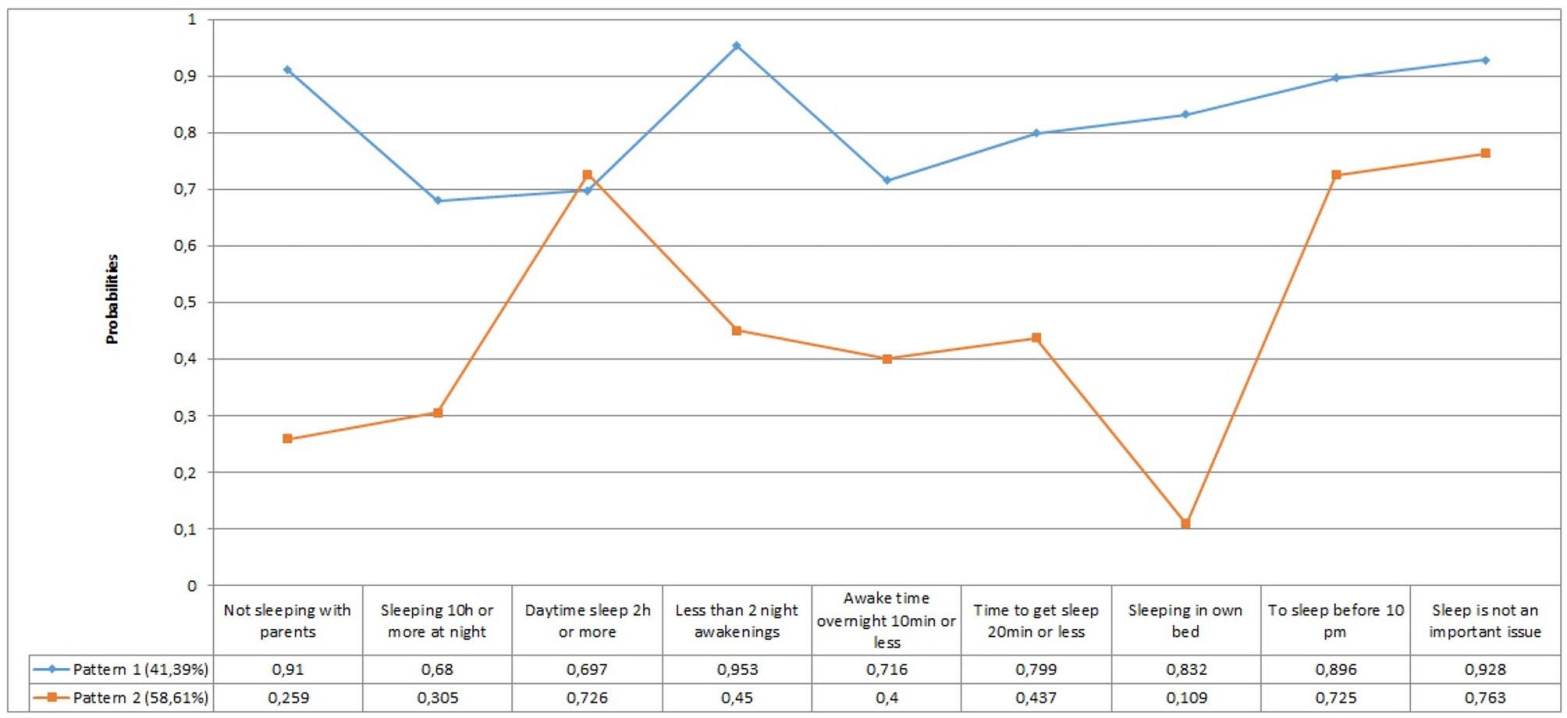
Graphical representation of the two patterns obtained through the latent class analysis. Pattern 1 is the “Good-Sleep Pattern”, and pattern 2 is the "Sleep-Disordered Pattern"

Latent class analysis (LCA) identified two sleep patterns (Fig. 1). Two hundred and four responses were completed to perform the LCA. Pattern one represented 41% of the sample. This pattern was characterized by children who do not sleep with their parents, sleep in their own bed, sleep for 10 h or more at night and 2 h or more during the day, have less than 2 awakenings overnight, staying less than 10 min awake, and takes less than 20 min to fall asleep. They went to bed before 10 PM, and sleep did not look like a problem for their parents. All these sleep characteristics are present in this pattern, with probabilities between 0.68 and 0.95. This pattern was called by us as "Good-sleep Pattern". Pattern two represented 59% of the sample. In this pattern, one or more characteristics of the "Normal Sleep Pattern" were present with a low probability (between 0.11 and 0.45). This meant that children in this pattern used to sleep with their parents for less than 10 h overnight, or had two or more awakenings during the night, or took more than 20 min to fall back to sleep or did not sleep in their own bed. This pattern was called by us as "Sleep-Disordered Pattern".

Analysis of the relationship between sleep patterns and other characteristics of parents and infants not included in the BISQ-E questionnaire, especially those related to co-sleeping and breastfeeding, are summarized in Table 3. Past or continued co-sleeping was significantly related to the "Sleep-Disordered Pattern" (p < 0.001). Also, in this same pattern, we found a higher probability of having difficulties in putting the baby to sleep at the time of answering the questionnaire (p < 0.001) as in the past (p = 0.015). With respect to the impact of breastfeeding, we also found differences between the two patterns. Infants who had breastfed were more likely to belong to pattern two. Finally, in pattern two ("Sleep-Disordered Pattern"), the percentage of parents who were worried about their child's sleep quality was higher (p < 0.001). Also, in pattern two, it was significant the rate of fathers (p = 0.029) or mothers (p = 0.032) that were concerned about their own sleep pattern.

**Table 3:** Summary of relationship between sleep patterns and other characteristics of parents and infants not included in the BISQ-E questionnaire

	**Total (n = 244)**	**Pattern 1 (n = 101)**	**Pattern 2 (n = 143)**	**P value**
**Sociodemographic variables**				
Mother's age (years) [mean (SD)]	35.4 (4.3)	34.8 (4.4)	35.9 (4.1)	0.058
Father's age (years) [mean (SD)]	38.0 (5.3)	37.6 (5.4)	38.4 (5.2)	0.277
Child's age (months) [mean (SD)]	20.2 (5.3)	21.2 (5.5)	19.6 (5.1)	0.018*
Child´s sex [n (%)] [Female]	113 (46)	42 (42)	71 (50)	0.213
Serious illness of the child [n (%)] [Yes]	13 (5.3)	3 (3)	10 (7)	0.168
Siblings of the child [n (%)] [Yes]	115 (47)	62 (61)	53 (37)	< 0.001**
**Variables related to the practice of co-sleeping**				
Have you practiced co-sleeping? [n (%)] [Yes]	164 (67)	44 (44)	120 (84)	< 0.001**
Is your child still sleeping in your bedroom? [n (%)] [Yes]	113 (46)	7 (6.9)	106 (74)	< 0.001**
Do you co-sleep in a specific crib? [n (%)] [Yes]	91 (46)	35 (54)	56 (42)	0.109
Age up to which co-sleeping has occurred [mean (SD)]	17.62 (11)	9.16 (6.7)	21.14 (11)	< 0.001**
Reasons to practice co-sleeping [n (%)] - Decided before birth - In response to the child's requests	85 (46.2) 99 (53.8)	28 (54) 24 (46)	57 (43) 75 (57)	0.191
Parents agree on the practice of co-sleeping? [n (%)] [Yes]	161 (93.1)	44 (94)	117 (93)	0.861
Does the child currently have difficulties sleeping? [n (%)] [Yes]	86 (35.2)	13 (13)	73 (51)	< 0.001**
Has the child had difficulty sleeping in the past? [n (%)] [Yes]	136 (55.7)	47 (46)	89 (62)	0.015*
**Variables related to breastfeeding**				
Have you given breast milk to your baby? [n (%)] [Yes]	216 (88.5)	84 (83)	132 (92)	0.027*
Until what age have you breastfed your baby? [mean (SD)]	12.26 (7.4)	8.57 (6)	14.68 (7.3)	< 0.001**
**Variables related to parental concerns**				
Are you worried about your child's sleep quality? [n (%)] [Yes]	79 (32.4)	14 (14)	65 (45)	< 0.001**
Does the father have sleep problems? [n (%)] [Yes]	39 (16.0)	10 (9.9)	29 (20)	0.029*
Does the mother have sleep problems?? [n (%)] [Yes]	30 (12.3)	7 (6.9)	23 (16)	0.032*

The relation between the categorical variables was calculated by applying the Chi-square test. Differences between the quantitative variables was calculated through a comparison of means using T-Student test. *0.001 < p < 0.05; **p < 0.001

## Discussion

Contrary to our hypothesis, the present findings indicate that co-sleeping during the first year of life was associated with the sleep pattern quality of young preschoolers. Specifically, co-sleeping resulted in being independently associated with a poor sleep quality pattern. Accordingly, parents who had practiced co-sleeping perceived their children's sleeping patterns as a severe problem. Compared to parents who had not practiced co-sleeping, they reported their infants slept fewer hours overnight, took longer to fall asleep, and were prone to fall asleep more often in their parents’ arms while eating or in their parent’s bed. One explanation for these results could be that sleeping with the baby may have induced an exaggerated perception of worse sleeping, and so of the BISQ-E items in the parents.

From an evolutionary point of view, co-sleeping reflects the importance of providing close and continuous physical contact within the dyad, which contributes to promoting an optimal infant’s development [8]. Moreover, evidence indicates that co-sleeping is associated with promoting successful breastfeeding [24]. Also, a recent paper states that adequate sleep at nighttime appears to be beneficial for children’s cognitive development [25]. Within this scenario, our study's findings apparently contradict, at least partially, the acknowledged beneficial effects associated with co-sleeping. Moreover, at univariate analysis, we also found that breastfeeding was associated with a poor sleep quality pattern.

However, considering that infants’ sleep patterns are dynamic and change over the first years of life [26], it could be speculated that the quality of the sleep pattern of preschoolers whose parents have practiced co-sleeping may improve over time. In line with this hypothesis, Abdul Jafar et al. conducted a cohort study to evaluate the association between breastfeeding and sleep trajectories from 3 to 54 months [27]. The authors found that breastfed infants showed increased night awakenings compared to formula-fed infants, with an attenuation of this finding from the age of 18 months, suggesting that the more frequent nocturnal wakefulness shown in breastfed infants may be transient.

The effect of co-sleeping during the first months of life on the subsequent sleep pattern of children has not been elucidated yet. In a study on 5-year-old children, Iwata et al. found that co-sleeping at that age did not negatively influence the sleep pattern, both for the child and their parents [1]. Mao et al. conducted a comparative study including babies from 3 to 15 months and found that babies who practiced co-sleeping had more nocturnal awakenings than babies who slept alone [11]. Fernandez-Miaja et al., in a study carried out on children under two years of age in a setting very similar to that of our study, reported a mean value of 10.4 h of night sleep and a mean value of 1.9 h of day sleep time, which are consistent with our findings [28]. On the contrary, the number of nocturnal awakenings found in our study was higher (2.0 vs 1.2). Persistent co-sleeping has also been associated with mother reports of marital and co-parenting distress and lower maternal emotional availability with infants at bedtime [29].

However, when addressing co-sleeping, it must be considered that co-sleeping is valued differently according to cultural backgrounds. In Western countries, infant solitary sleeping is a pursuit aiming at the early achievement of separateness and independent functioning [30]. Accordingly, Mindell et al., in a study including almost 30,000 children, reported that in Asian countries, children share a room and bed with their parents much more frequently than in Caucasian countries [31]. Furthermore, in China, most parents believe that co-sleeping means security, happy family intimacy, and a child’s long-term emotional development [32].

According to our results, a higher percentage of preschoolers fell asleep in their parent’s arms, in their parent’s bed, or eating when they had practiced co-sleeping. In contrast, they fell asleep in their cot or mattress more often if they had not practiced it. We can argue that co-sleeping induces physical dependence in babies while going to sleep, but more research is needed on this association.

Few studies have used latent class analysis concerning sleep habits in preschool children. In a Portuguese cohort of 1092 children, Goncalves et al. identified two behavior patterns at 4 years of age. The pattern with the most sleep difficulties is related to the fact that the mother raises the child without a partner and that the child has no siblings or is not attending daycare [33]. Levenson et al. assess the sleep pattern of children aged 2–5 years, using latent class analysis to find an optimal four-class solution. They conclude that those with a better sleep pattern than their parents have fewer psychopathological difficulties [34]. None of these studies assess the effect of co-sleeping or breastfeeding on sleep patterns. None of these studies evaluate the impact of co-sleeping or breastfeeding on sleep patterns.

Almost all parents who responded to our survey reported that their children had no illness that particularly worried them. However, we consider that babies with some special needs could practice co-sleeping, and consequently, their parents may perceive more difficulties sleeping. This would, however, be the subject of another research design. In this study, we have also not considered specific genetic or family variables regarding sleep that can undoubtedly negatively influence it.

Our study has had several limitations. The first limitation of our research is that the response rate could not be calculated. Secondly, our results have been obtained through a survey based on parents’ perceptions. However, BISQ-E is a well-established, validated instrument to assess sleep quality. Third, since the questionnaire was distributed through schools or pediatric clinics, people from different sociodemographic areas of Spain could potentially have not been reached. Fourth, given the cultural differences between countries regarding infant sleep, it may be difficult to extrapolate our results since what is suitable for some parents, such as sharing a bed beyond one year of age, may be understood as inappropriate for other cultures or frustrating. Fifth, it´s a fact that the sleep pattern of the population studied changes over time from one to two and a half years of age; however, the authors consider that the difficulties do not vary excessively, focusing, for example, on the need to feel accompanied, the number of awakenings or the time taken to fall asleep. Sixth, no minimum number of months of co-sleeping practice was set to answer this question positively; however, language-wise, it is understood that this had been common practice for months. Seventh, in the questionnaire, we asked if there were any illnesses that might be of concern to parents. However, we did not specifically ask about specific chronic illnesses. Although the percentage of parents who responded positively to this question was small, it is possible that a percentage of them had certain significant chronic illnesses that may have affected the baby's sleep and, therefore, our results. Thus, these results should be taken with caution. Future research endeavors, particularly those employing a prospective and more rigorous study design, are anticipated to provide a more definitive resolution to our research inquiry.

In summary, our study has identified that co-sleeping during the first year of life is associated with young preschoolers’ sleep patterns. Further, longitudinal studies are needed to gain further insight into the contribution of co-sleeping on the long-term development of sleep pattern quality. With our results, we do not propose limiting the practice of co-sleeping in parents who decide to do so, as it has proven benefits; however, we want to draw attention to the potential subsequent effects on the sleep pattern. If future studies confirm this association, parents should be aware of it and know how to improve their children's future sleep patterns.

### Supplementary Information

Below is the link to the electronic supplementary material.

Supplementary file1 (PDF 179 KB)

Supplementary file2 (PDF 109 KB)

## Data Availability

The datasets generated during and/or analyzed during the current study are available from the corresponding author upon reasonable request.
