# Correction to: Association between co-sleeping in the first year of life and preschoolers´ sleep patterns

**DOI:** 10.1007/s00431-024-05533-3

**Published:** 2024-03-26

**Authors:** Felipe Garrido, Juan-Luis González-Caballero, Pilar García, Maria-Lorella Gianni, Silvia Garrido, Lucía González, Verónica Atance, Genny Raffaeli, Giacomo Cavallaro

**Affiliations:** 1 Department of Pediatrics, Clínica Universidad de Navarra, Calle Marquesado de Santa Marta, Madrid, 28227 Spain; 2 Department of Statistics and Operations Research, Faculty of Medicine, University of Cadiz, 11003 Cádiz, Spain; 3 Jerome Lejeune Institute, Madrid, 28227 Spain; 4 Department of Clinical Sciences and Community Health, Università degli Studi di Milano, 20122 Milan, Italy; 5 Neonatal Intensive Care Unit, Fondazione IRCCS Ca’ Granda Ospedale Maggiore Policlinico, 20122 Milan, Italy

**Correction to: European Journal of Pediatrics** 10.1007/s00431-024-05429-2

The wrong Supplementary file was originally published with this article; it has now been replaced with the correct file.

The original article has been corrected.

### Supplementary Information

Below is the link to the electronic supplementary material.

Supplementary file1 (PDF 178 KB)

Supplementary file2 (PDF 109 KB)

