# Supplementary material for: Association between co-sleeping in the first year of life and preschoolers´ sleep patterns
Source: Eur J Pediatr. 2024 Feb 14;183(5):2111–9. doi: 10.1007/s00431-024-05429-2 (PMC11035441; doi:10.1007/s00431-024-05429-2)
Supplement: Supplementary file 1 — Supplementary file1 (PDF 179 KB) [file 431_2024_5429_MOESM1_ESM.pdf]

Effects of sleep habits during breastfeeding on subsequent sleep quality during early preschool years

1. Presentation of the survey

Is your child between 12 and 30 months old?

We invite you to help us in this research and thank you for your participation!

Before deciding whether you wish to participate in this study, it is important that you carefully read the information provided below.

Previous studies have found that 25-50% of the paediatric population may have some kind of sleep disorder during their childhood. Researchers want to know more about the sleep of preschoolers and their parents, and how the habits acquired during breastfeeding have influenced it.

From question 23 to 32 you will answer the questions of the BISQ-E questionnaire that assesses the quality of your child's sleep, ONLY of your child between 12 and 30 months of age, and we need you to answer it describing what has happened in the LAST TWO WEEKS (Cultural adaptation of the BISQ-E; Adaptation and study of metric properties of a sleep assessment questionnaire for infants and preschoolers: Casanello et al; An Pediatr (Barc). 2018;89(4):230-237).

When we ask, in the second part of the survey about the mother's sleep difficulties, we refer to what has happened in the LAST MONTH (The Spanish version of the Pittsburgh Sleep Quality Index; Macias et al. Informaciones psiquiátricas; 1996; 146 (465-72)).

You should be aware that your participation in this study is voluntary.

Due to the design of the study, we do not collect personal data about you or your children. The researchers will not be able to access the identity of the mothers who respond to the questionnaire. Each response will be assigned an automatic code by the response collection system. All data obtained from your participation in the study will be stored in a secure, restricted-access location. The Data Protection Act and other applicable laws will be followed throughout the process.

Information sheet for parents

Would you like to participate freely in this study?

- Yes
- No

Effects of sleep habits during breastfeeding on subsequent sleep quality during early preschool years

2. General aspects of your family

In these first questions we want to know something about your family, habits, and about the child we are going to find out about his/her sleeping habits.

Can you tell us the age of the mother (in NUMBER please, e.g. 34)?

Can you tell us the age of the father (in NUMBER please, e.g. 34)?

What day is today (DD/MM/YYY)?

What is your child's date of birth (DD/MM/YYYYY)?

What is your bond with the child?

- Mother
- Father

What is the sex of the child?

- Male
- Female
- Other: specify

Does your child have or has your child had a serious illness that has caused excessive parental concern?

- Yes
- No

How many children live together at home with the child whose sleep habits we are assessing?

What is the order of your child's siblings, if any?

- It is the largest
- It is intermediate
- It is the small
- No siblings

Is your child still sleeping in his/her bedroom?

- Yes
- No

If your child no longer sleeps in his or her room, can you tell us how many months old he or she moved to his or her own bedroom to sleep?

Are you CURRENTLY worried about your child's sleep?

- Yes
- No

Are you CURRENTLY having difficulty getting your child to sleep?
- Yes
- No

Have you in the PAST had difficulty getting your child to sleep?
- Yes
- No

Have you ever practiced or do you practice BEDMATCH EVERY NIGHT or VERY HABITUALLY with your child? (BEDMATCH is defined as the practice of sharing the bed of adults, usually parents with their children, during sleeping periods. HABITUAL COUCHING is defined as the practice of bed sharing at least 3 times a week):
- Yes
- No

If you practise or have practised COLECHO, have you used a bed / cot specifically designed for COLECHO?
- Yes
- No

If you have slept with your child, until what age (in MONTHS) did you usually sleep with your child? (In case you still sleep with your child, please indicate the age until which you plan to continue sleeping with your child, in MONTHS) (Please answer with a number, e.g. "8" if you mean 8 months).

If you have practised COLECHO, were you thinking of practising it before the birth or did you start practising it as a RESPONSE to the baby's NIGHT TIME REQUIREMENTS?
- Yes, I was planning to do it before my baby was born.
- No, we have practised it as a response to the baby's requirements.

In case of co-sleeping, do both parents agree to co-sleeping?
- Yes
- No

Has the child whose sleep pattern we are assessing been breastfed?
- Yes
- No

If yes to the previous question, until what age (IN MONTHS) did you breastfeed exclusively or with other foods?

Did the father usually have problems falling asleep before the birth of his child?
- Yes
- No

Did the mother usually have problems falling asleep before the birth of her child?
- Yes
- No

3. Cultural adaptation of the Brief Infant Sleep Questionnaire to Spanish (BISQ-E). AnPediatr(Barc).2018;89(4):230-237

Please select the answer you think best describes your family's habits and customs during the last TWO WEEKS.

Where does your child CURRENTLY sleep?

- Sleeps in the cot/bed, in a separate bedroom
- Sleeps in cot / bed, in parents' room
- Sleeps in crib/bed, shared with another sibling
- Sleeps in bed with parents
- Please specify

What is the position in which the child CURRENTLY sleeps most of the night?

- Face down
- Sideways
- Face up
- It does not have a defined position

How long does the child sleep at NIGHT (between 8 p.m. and 7 a.m.)?

Hours	
Minutes	

How long does the child sleep during the DAY (between 7 a.m. and 8 p.m.)?

Hours	
Minutes	

How many times does your child wake up during the night (on average)?

How much time does the child spend awake during the night (from 8 p.m. to 7 a.m.)?

Hours	
Minutes	

How long does it take for the child to fall asleep after going to bed?

Hours	
Minutes	

How do you get the child to fall asleep?

- While eating
- Rocking in a cot or similar.
- Rocking him in my arms
- Holding him in your arms without rocking
- In your cot/bed, in separate room
- In their cot/bed, in the parents' room

- In the same bed as the parents
- Other (please specify)

On weekdays, around what time do you usually fall asleep at night?

hh mm -

Hour a. m./p. m.

Do you consider the child's sleeping habits a problem?
- Yes, a very serious problem
- Yes, although it is a minor problem
- No, it is not a problem
