# Supplementary material for: Association between co-sleeping in the first year of life and preschoolers´ sleep patterns
Source: Eur J Pediatr. 2024 Feb 14;183(5):2111–9. doi: 10.1007/s00431-024-05429-2 (PMC11035441; doi:10.1007/s00431-024-05429-2)
Supplement: Supplementary file 2 — Supplementary file2 (PDF 109 KB) [file 431_2024_5429_MOESM2_ESM.pdf]

Universidad de Navarra

PATIENT INFORMATION SHEET

Study title: **Effects of sleep habits during infancy on subsequent sleep quality during early preschool age.**

Principal Investigator: Pilar M García Fernández

Centre: Clínica Universidad de Navarra. Department of Paediatrics.

Madrid. First of all, we thank you for your interest in participating in this

study.

We invite you to participate in a research study on the influence of sleep habits during breastfeeding on the subsequent quality of sleep during the school years. The study has been approved by the Research Ethics Committee of the University of Navarra. Before deciding whether you wish to participate in this study, it is important that you understand why this research is interesting, what your participation will involve, how your information will be used and its possible benefits, risks and discomforts.

Please take the time to read the information provided below carefully.

What is the reason for the study? To find out whether sleep habits during breastfeeding influence preschool age.

How will the study be done? Using a questionnaire specifically designed by the researchers and which includes some validated tools for measuring the quality of sleep in both the child and the mother.

You should be aware that your participation in this study is voluntary, and that you may decide not to participate or to change your decision and withdraw your consent at any time, without altering your relationship with your doctor/nurse.

Should you decide to withdraw from the study, you may do so by allowing the use of the data collected so far for the purpose of the study or, if you wish, deleting all data from the computer files.

Who can participate? The study is open to all parents who wish to answer the questionnaire, who are parents of a preschooler aged between 12 and 30 months, and who have accessed the questionnaire through information provided by their paediatricians, doctors or nurses.

Your participation in the study is limited to answering the questionnaire provided by your paediatrician, doctor or nurse. We do not collect any personal data from you, except the IP (internet protocol address) from which you answer, which will be duly guarded by the principal investigator.

No financial compensation of any kind is foreseen during the study. Funding: No

external funding is available for this study.

All personal data, including clinical data, will be processed in accordance with current data protection laws, in particular the RGPD and the LOPD.

The Data Controller (CUN), in compliance with Regulation (EU) 2016/679 of the European Parliament and of the Council of 27 April 2016 on the protection of individuals with regard to the processing of personal data and on the free movement of such data, hereinafter referred to as GDPR, informs you that, if you participate in this survey, the answers provided will be processed by the team.

researcher to draw conclusions from the Project. The data will also be accessible to health authorities and members of the ethics committee if deemed necessary.

It will not be possible to identify you from any communications that may be generated by this study.

You are responsible for the veracity and correctness of the data you provide us with and you have the right to exercise your rights of access, rectification, suppression, limitation of processing, portability and opposition of your data in accordance with the provisions of data protection regulations. To exercise these rights, you should write to the Data Protection Delegate of (CUN/UN), or to the following postal address Departamento de Pediatría, Clínica Universidad de Navarra, Marquesado de Santa Marta 3, Madrid or to the e-mail address fgarridom@unav.es , in either case you should attach a photocopy of your national identity card or equivalent.

In the event that you do not agree with the processing carried out by our Entity or consider that your rights have been violated, you have the right to file a complaint with the Spanish Data Protection Agency.
